# Examining the Inverted U-Shaped Relationship Between Benevolent Leadership and Employees’ Work Initiative: The Role of Work Engagement and Growth Need Strength

**DOI:** 10.3389/fpsyg.2022.699366

**Published:** 2022-05-05

**Authors:** Huan Li, Saisai Sun, Pu Wang, Yating Yang

**Affiliations:** ^1^School of Business and Management, Shanghai International Studies University, Shanghai, China; ^2^Linyang Group, Shanghai, China

**Keywords:** benevolent leadership, work engagement, work initiative, growth need strength, inverted-U effect

## Abstract

Benevolent leadership is generally considered to be beneficial for work initiative. However, based on social exchange theory, this paper explores an inverted U-shaped relationship between benevolent leadership and work initiative. Using a multilevel structural equation model that analyzed the data from 596 employees and 139 supervisors in multiple technology companies, our findings show that benevolent leadership had an indirect, negative curvilinear relationship with work initiative *via* work engagement at both the individual and team levels. Furthermore, we also indicate that growth need strength moderates the positive relationship between benevolent leadership and work engagement at the individual level. Theoretical and practical implications and future research directions are discussed.

## Introduction

For decades, studies have shown the prevalent influence of benevolent leadership across various cultural contexts (Pellegrini and Scandura, 2008; Wang and Cheng, 2010; Karakas and Sarigollu, 2012; Lin W. et al., 2018; Wang A. C. et al., 2018). Benevolent leaders tend to demonstrate individualized, holistic concern for their followers’ well-being (Farh and Cheng, 2000, p. 94). In return for the leader’s benevolent behavior, followers show gratitude and desirable behaviors, which bring beneficial results to their organizations because of their sense of obligation and reciprocity (Lin W. et al., 2018). Given the important role of encouraging followers’ trust, obligation, gratitude, and sense of debt, a growing body of researches have been launched to explore the impact of benevolent leadership in organizations. For instance, prior researches found that benevolent leadership had a favorable impact on a majority of outcomes, including innovative behavior (Wang and Cheng, 2010; Lin W. et al., 2018), work initiative (Xu et al., 2018), job satisfaction (Cheng et al., 2002), psychological well-being (Erkutlu and Chafra, 2016), and employee voice (Zhang et al., 2014).

However, some studies have also discovered the potential downside of benevolent leadership (Li et al., 2018; Shaw et al., 2020). For example, based on the too-much-of-a-good-thing (TMGT) effect, Li et al. (2018) asserted that benevolent leadership had an inverted U-shaped (negative curvilinear) relationship with team performance through team action processes, which led the scholars to gain insight into understanding how and when benevolent leadership had a negative impact. The TMGT effect stated that a beneficial antecedent variable would be detrimental to outcomes when it exceeded the inflection point (Pierce and Aguinis, 2013). In line with this, Li et al. (2018) recommended that future studies should probe whether other intervening mechanisms such as demotivation might also work when the TMGT effect occurred. As demotivation is the opposite of motivation,^1^ and work engagement refers to a positive motivational state and attitude that is characterized by absorption, vigor, and dedication in the workplace (Maslach et al., 2001; Schaufeli et al., 2002; Mauno et al., 2007), it makes us doubt whether benevolent leadership has a negative curvilinear relationship with employees’ work engagement.

Furthermore, we also posit that there is an inverted-U relation between benevolent leadership and work initiative, which is inconsistent with the prior study. Prior research noted that subordinates who perceived high benevolent leadership behavior tended to feel a strong sense of gratitude (Lin W. et al., 2018), thereby resulting in high work initiative (Xu et al., 2018). Personal initiative is defined as “a behavior syndrome resulting in an individual taking an active and self-starting approach to work and going beyond what is formally required in a given job” (Frese et al., 1997, p. 140; “work initiative,” hereafter). Considering the potential negative curvilinear relationship between benevolent leadership and work engagement, we argued beforehand, and the fact that employees’ work engagement means individuals will have a high level of energy, enthusiasm, and persistence (Schaufeli et al., 2002, 2008), which may increase their work initiative in the organizational environment (Salanova and Schaufeli, 2008; Lisbona et al., 2018); we suggest that excessive benevolent leadership may decrease work initiative as a result of reducing their work engagement. As such, we argue that benevolent leadership appears to have a positive association with employees’ work initiative up to a point after which excessive benevolent leadership may hinder work initiative.

To clarify these issues, our main purpose is to examine whether benevolent leadership has an inverted-U relationship with work engagement, while at the same time affecting work initiative. This purpose called for two precautions. First, scholars contended that work context might influence team employees in the same way, thereby forming the shared and positive team work engagement (Costa et al., 2014), yet, empirical evidence is scant. Besides, previous researches presumed that there might be difference in the influence of work engagement at team and individual level (Tims et al., 2013; Mäkikangas et al., 2016). Therefore, our work aims to identify whether the individual-level effect is distinguishable from team-level effect (Kenny and Voie, 1985; Raudenbush and Bryk, 2002) and demonstrate whether there are team- and individual-level inverted-U relationship between benevolent leadership and work initiative through work engagement. Second, individual factors can influence subordinates’ psychological state (Tiegs et al., 1992). Due to the fact that the effect of benevolent leadership might vary depending on the existence of individual factors (Wang and Cheng, 2010), we investigate the possible moderating roles of growth need strength in the relationship between benevolent leadership and employees’ psychological mechanism at the individual level. Growth need strength as an element of followers’ characteristics reflects individuals’ desire to grow and develop within their jobs (Shalley et al., 2009), which may play a significant role in how employees respond to their leaders’ behavior (Algattan, 1985; Wang, Y. et al., 2018). Besides, previous studies have illustrated that growth need strength would strengthen motivation at work (e.g., Oldham, 1976; Evans et al., 1979; Zargar et al., 2014). As work engagement is an indicator of motivation in the workplace (e.g., Salanova et al., 2005; Salanova and Schaufeli, 2008), we expect to extend our understanding of how benevolent leadership influences work engagement under the employees holding different growth need strengths.

Our research provides several contributions. First, prior work has mainly focused on desirable aspects of benevolent leadership in facilitating work engagement (Tuan, 2018; Xu et al., 2018). In contrast to these findings, building on the social exchange theory, we investigate effects of benevolent leadership that may have less beneficial and unintended consequences for subordinates. Second, in addition to team action processes, which has been served as an underlying mechanism that explains the inverted-U relationship between benevolent leadership and favorable outcomes (Li et al., 2018), we now understand the influence of work engagement on work initiative. By examining the mediating role of work engagement, our research posits the motivational mechanism that may reveal why excessive benevolence will induce undesirable outcomes (i.e., low work initiative) in the organization. Third, by introducing growth need strength as a moderator, we contribute to the expansion of benevolent leadership literature not only to provide a comprehensive understanding of the relationship between benevolent leadership and subordinates’ work initiative, but also to provide implications for practitioners in trying their best to maximize employees’ work initiative.

## Theoretical Framework and Hypotheses

### Benevolent Leadership

Benevolent leadership refers to leader behaviors that demonstrate individualized, holistic concerns for subordinates’ personal, and family well-being (Farh and Cheng, 2000, p. 94), which is originated from the ideal balance of the five relationships in Confucianism (such as a benevolent ruler with his loyal ministers, or a kind father with his filial sons). Benevolent leaders will provide job security, guide career development, and even protect workers who make grave mistakes in the work domain (Farh and Cheng, 2000). Outside the work, they further show overall concern, such as providing followers with whatever they need, and even manifest interest in their personal lives (Cheng et al., 2000).

According to Farh and Cheng (2000), benevolence from benevolent leadership is spontaneous, although there is no compulsory institutional force to require leaders to engage in benevolent behaviors toward their subordinates. Subordinates will feel indebted to this benevolence by showing gratitude, remaining personal loyalty, and working hard in return (Farh and Cheng, 2000). Thus, benevolent leadership is a widespread management phenomenon rooted in the Chinese traditional culture (Cheng and Wang, 2014; Lin W. et al., 2018), which has been shown as an effective leadership influencing on a variety of followers’ beneficial results (e.g., Chan, 2017; Gumusluoglu et al., 2019).

### Work Engagement

Work engagement is defined as an affective-motivational, work-related state of mind in employees that is characterized by vigor, dedication, and absorption (Schaufeli et al., 2002). This definition is in line with the conceptualization of Rothbard (2001), which also categorize it as motivation. Vigor is defined as having high energy and mental resilience when they work, willing to make efforts for job, as well as persisting in difficult context; dedication is defined specifically as having strong sense of involvement, enthusiasm, pride, and challenge in their work; absorption refers to concentrating on their work whereby time passes quickly and being difficult to detach from work (Schaufeli et al., 2002, p. 74). Studies have demonstrated that job resource (e.g., supervisor support, job control, and job autonomy) was positive associated with work engagement (Salanova et al., 2005; Bakker et al., 2007; Mauno et al., 2007). Besides, employees who are engaged in work are more likely to find meaning at work (e.g., Macey and Schneider, 2008), allocate personal resource to performance (e.g., Wang et al., 2015), and experience motivational fulfilment to acquire initiative (e.g., Hakanen et al., 2008).

### Work Initiative

Work initiative is originated from action theory, which is a specific form of proactive behavior, and is based on developing a fuller set of goals that go beyond the scope of formal requirements in the job and by being proactive, which is understood to include extra-role performance (Frese et al., 1996, 1997; Den Hartog and Belschak, 2007). More specifically, “work initiative (1) is consistent with the organizational goal, (2) has a long-term focus, (3) is goal-directed and action-oriented, (4) is persistent in the face of barriers, and (5) is self-starting and proactive” (Frese et al., 1996, p. 38).

There is the similarity between work engagement and work initiative; they both mean that individuals are greatly engaged in their work (Lisbona et al., 2018). Nevertheless, there are also differences in the concepts of work engagement and work initiative. For example, work engagement is the motivational state that may develop initiative (Schaufeli et al., 2002), but not the same as work initiative behavior. Besides, willing to be absorbed in work is not the same as continuing the work behavior despite of difficulties (Lisbona et al., 2018).

### Benevolent Leadership, Work Engagement, and Work Initiative

#### Individual-Level Relationships

Several studies have implied that benevolent leadership is relevant in influencing employees’ work engagement (Tuan, 2018; Xu et al., 2018). For instance, according to the research by Xu et al. (2018), followers will induce their positive emotion because of their benevolent leaders’ supportive, considerate, helpful, and caring behavior. In addition, benevolent leaders caring about their subordinates’ achievement inside as well as outside workplaces will engender positive affective state (Tuan, 2018). These researches suggested the possibility of the presence of relationship between benevolent leadership and work engagement.

However, the relationship between benevolent leadership and work engagement may not be as simple as the “the more, the better” conclusion indicates. As mentioned above, there is empirical evidence that excessive benevolent leadership has negative consequences (Li et al., 2018). Indeed, Farh and Cheng (2000) also noted that benevolent leadership might conflict with modern values when it is put into practice. For instance, benevolent leadership may bring about dissatisfaction among employees by allocating resources to subordinates based on need rather than fairness (Cheng et al., 2000), in turn reducing followers’ work engagement (Maslach and Leiter, 2008). In addition, benevolent leaders do not punish employees after they make mistakes (Farh and Cheng, 2000); whether this behavior is reasonable remains uncertain.

We attempt to use the too-much-of-a-good-thing effect to account for the potential negative impact of excessive benevolent leadership on work engagement (Pierce and Aguinis, 2013). The TMGT effect argues that when a beneficial antecedent variable reaches a certain inflection point, the relationship with the outcome variable is no longer linear, which means that predictive relationship between the antecedent variable and the outcome variable should not exceed this inflection point because exceeding it might not bring additional benefits or even cause adverse consequences (Pierce and Aguinis, 2013).

The TMGT effect can explain some of the phenomena in the management literature that contradicts common sense (Pierce and Aguinis, 2013). For example, Mo et al. (2019) found that ethical leaders who overemphasized the need for moral behavior made their subordinates refrain from innovative behavior. In addition, Li et al. (2018) explained that benevolent leadership harmed team performance because excessive benevolence would cause supervisors to spend less resources and time to concentrate on work task. The philosophical tenet behind the TMGT effect is that doing too much of a good thing will become a bad thing, which is consistent with the “the golden mean” advocated by traditional Confucianism (Pierce and Aguinis, 2013). Therefore, we use the TMGT effect to speculate on the possible nonlinear effect of benevolent leadership that is also rooted in Confucianism (Li et al., 2018).

A lack of benevolence is harmful to the team to a large extent because team members do not get enough good things (Li et al., 2018, p. 372), and moderate level of benevolence will induce team members being obligated and reciprocal to repay (Gouldner, 1960; Farh and Cheng, 2000; Li et al., 2018; Lin W. et al., 2018). Thus, we argue that benevolent leadership will develop higher work engagement when the degree of benevolence increases from a low level to an optimal intermediate level. First, benevolent leaders will demonstrate individualized care and treat their followers as family members (Farh and Cheng, 2000), which will arouse their followers’ feelings of obligation to the role expectations (Farh and Cheng, 2000; Wang and Cheng, 2010). Since subordinates tend to repay their leaders and meet leaders’ expectations in the social exchange process (Blau, 1964; Farh and Cheng, 2000), those employees will increase their work engagement when understanding their supervisors’ high-performance orientation in organizations (Tanskanen et al., 2019). Second, followers can develop a high level of perception of support because of leaders’ benevolence. After benevolent leader assists subordinates when they are in emergencies, subordinates will generate strong sense of support from their leaders (Cheng et al., 2000; Chan, 2017). With perceived support, employees will increase their sense of belonging and improve their work engagement afterward (Schaufeli et al., 2008). Moreover, prior studies have recognized that leadership, as an important part of the work environment, influences work engagement owing to leaders’ social support (Rabinowitz and Hall, 1977; Chen et al., 2020). As such, we expect that benevolent leadership is positively related to work engagement when benevolent leadership is at a low to a modest level.

However, as the TMGT effect summarizes, increasing effective leadership will lead to positive outcomes up to an inflection point, after which they cause detrimental outcomes for followers and their organizations (Pierce and Aguinis, 2013). We suggest that when the level of benevolence reaches a certain critical point, as the level of benevolence further increases, work engagement may decrease. Based on social exchange theory, the norms of reciprocity stressed that individuals who did not comply rules would be punished (Cropanzano and Mitchell, 2005). In a heterogeneous population, the selfish free riders often benefit the most while others pay the cost of cooperation (Guala, 2012; Price and Vugt, 2014). Thus, scholars emphasized an effective approach to stabilize the reciprocal process, which was to punish people who were unhelpful in the teams (Panchanathan and Boyd, 2004; Cropanzano and Mitchell, 2005; Guala, 2012). From this point, taking punitive action is to ensure employees’ reciprocity and cooperation (Bowles and Gintis, 2004). However, as stated above, excessively benevolent leaders protect their subordinates and avoid punishing them when they make serious mistakes (Farh and Cheng, 2000), which may signal to shirkers that they will be free from penalties and reap substantial profits even if they do nothing (Bowles and Gintis, 2004; Rockenbach and Milinski, 2006). Compared with harsh punishment conditions, subordinates can alleviate the concern about the risks of misbehaviors in organizations if the supervisor omits punishment (Trevino and Ball, 1992; Shaw and Liao, 2020). This might induce employees to deem that they are less likely to have serious consequences and to consider that their leaders find it acceptable for shirkers to have low work engagement (Hinkin and Schriesheim, 2008; Ghorpade et al., 2016; Shaw et al., 2020). Based on the above discussion, we propose the following:

*Hypothesis 1a*: At the individual level, benevolent leadership has an inverted U-shaped relationship with work engagement.

As pointed out above, work initiative was more behavioral than work engagement was (Hakanen et al., 2008; Lisbona et al., 2018), thus, we argue that with enhanced work engagement, there is potential for facilitating the development of work initiative. Work engagement itself is conceptualized as a positive state of employee motivation that is associated with discretionary effort. According to Frese et al. (1996, p. 41), motivational and skill development processes primarily influence initiative. If employees have sufficient potential to maintain and develop “effectance,” as well as intrinsic motivation, they will redefine the motivation of work in an enlarged extra-role sense and then demonstrate personal initiative (White, 1959; Frese et al., 1996). Moreover, positive emotion (i.e., work engagement) broadens employees’ momentary thought-action repertoires and builds up their enduring personal resources, which sparks a willingness to figure things out and is the kind of initiative that produces new ideas and novel solutions for the long term (Hakanen et al., 2008). In support of our argument, Bailey et al. (2017) indicated that extra-role performance, including work initiative, is possibly the outcome of work engagement. Hakanen et al. (2008) proposed that work engagement was positively associated with work initiative (Hakanen et al., 2008). Based on the above discussion, we propose the following:

*Hypothesis 2a*: At the individual level, benevolent leadership has an indirect, inverted U-shaped relationship with work initiative through work engagement.

#### Team-Level Relationships

Next, we further explore whether benevolent leadership has an inverted U-shaped indirect relationship with team work initiative through team work engagement. Generally speaking, research at the individual level tends to emphasize on the vertical dyad leadership style between supervisors and their followers, while researches at the team level are more likely to stress the average leadership between the supervisors and all team members (Cheng and Wang, 2014). Therefore, we not only focus on the impact of benevolent leaders on employees’ outcomes at individual level, but also investigate whether benevolent leaders have impact on teams’ outcomes. Although previous literature conceptually argued that work environment and events would influence team work engagement in a similar way (Costa et al., 2014), regrettably, empirical studies on team work engagement are still quite limited.

Team work engagement is defined as a shared, positive, motivational emergent state of work-related well-being (Costa et al., 2014). Team members with high levels of work engagement will communicate with each other about the importance of their work and express the difficulty of detaching themselves from work. As Costa et al. (2014) stated, a leader who is capable of setting clear goals will lead to an increase in the same team’s level of engagement because an employee under the influence of his/her supervisor will interact with team members.

A minority of shirkers will force positive reciprocators to low levels of cooperation in the end (Guala, 2012), we argue that the relationships between benevolent leadership and work engagement, at the team level under the guidance of the same supervisor, are maintained in parallel (Giolito et al., 2020). Although there is no research noting that benevolent leaders are team-oriented, the leaders indeed worked with followers to create a shared common vision (Karakas and Sarigollu, 2012). This argument is supported by a study conducted by Gumusluoglu et al. (2017), who reported that team-level benevolent leadership is positively related to team creativity *via* the mediating role of team identification. Moreover, Li et al. (2018) validated that benevolent leadership has a negative curvilinear effect on performance at the team level. Based on the above discussion, we propose the following:

*Hypothesis 1b*: At the team level, benevolent leadership has an inverted U-shaped relationship with work engagement.

We also argue that team work engagement might be positively correlated with team initiative. Brav et al. (2009) defined team-level work initiative as groups’ proactive approach to work associated with work activities beyond the stipulated task to achieve meaningful change. They considered that team motivation would stimulate team members to enhance their team initiative. Given that team work engagement is a team motivational state of work-related well-being, we hypothesize as follows:

*Hypothesis 2b*: At the team level, benevolent leadership has an indirect, inverted U-shaped relationship with work initiative through work engagement.

### Moderating Role of Growth Need Strength

Although we posit that benevolent leadership have an impact on team work engagement in a similar way, followers’ work engagement varies under the same benevolent leaders because of individual trait difference (Sonnentag, 2003). For example, those who strongly desire learning and self-development may be more likely to possess positive motivation state (Oldham, 1976; Houkes et al., 2001). Individuals vary on growth need strength, or the degree to which an employee values personal learning, growth, and development opportunities in their job (Hackman and Oldham, 1980; Tiegs et al., 1992). Research has shown that it is a potential job motivation variable that regulate the relationship between work context and work output as well as psychological state (Tiegs et al., 1992; Shalley et al., 2009; Oldham and Hackman, 2010). More specifically, employees with high growth need strength tend to attach importance to personal development and learning and engage more fully in job activities (Brown, 1996). However, those with lower growth need strength are less likely to handle complex and challenging tasks and even have a negative attitude toward the organization (Hackman, 1980). Hence, we propose that growth need strength is an individual factor that may regulate the relationship between benevolent leadership and work engagement.

Benevolent leadership will create an interdependent and cooperative relationship between leaders and their subordinates (Pellegrini et al., 2010; Chan and Mak, 2011). This cooperation aims to enable leaders and employees to meet each other’s needs, thereby increasing effectiveness and enjoyment at work (Jacobs, 1973; Graen et al., 1986). Cooperation with leaders could be viewed as a challenge to employees, and employees could choose whether to accept this challenge or not (Graen et al., 1986). Thus, employees with a high level of growth need strength would voluntarily take on this stimulating challenge (Spector, 1985) and have high intrinsic motivation to further facilitate work engagement under benevolent leadership. In contrast, it could be expected that employees with a low level of growth need strength would choose to give up growth opportunities and not cooperate with their leaders, resulting in low work engagement (Graen et al., 1986).

Additionally, benevolent leadership reduces work barriers, provides support to employees under pressure, and meets their needs according to employees’ different requests (Karakas and Sarigollu, 2012), and the reason leaders treat employees benevolently is for mutual reciprocity (Farh and Cheng, 2000). Hackman and Oldham (1976) considered that individuals who have high growth need strength are motivated to find and meet the demands of the situation. This means that growth need people are able to realize that the reason for leaders’ individualized care is that they need their followers to return, and, in turn, employees will respond with positive motivations in work (Saavedra and Kwun, 2000).

Some empirical evidence supports growth need strength as a potential moderator. Wofford et al. (2001) found that high growth need strength strengthens the positive relationship between transformational leadership and satisfaction with supervision. Moreover, Wang, Y. et al. (2018) argued that growth need strength is a moderator in the relationship between humble leadership and psychological capital and reported that high growth need strength enhances the relationship between humble leader behavior and followers’ psychological capital. These studies support the notion that growth need strength may constitute a moderator of the correlation between leadership and employees’ psychological mechanism (Tiegs et al., 1992). As such, we propose the following hypothesis:

*Hypothesis 3*: At the individual level, growth need strength moderates the relationship between benevolent leadership and work engagement such that the influence of benevolent leadership is more positive when an individual’s level of growth need strength is high than when it is low.

Last, the above-mentioned prediction implies first-stage moderation (Hayes, 2015), in which growth need strength moderates the indirect relationship between benevolent leadership and work initiative through work engagement. Employees with high levels of growth need strength will achieve higher work engagement under benevolent leadership; thus, they are inclined to exert more initiative at work. On the contrary, those with low levels of growth need strength will be less likely to raise their work initiative, since they will not cooperate with their benevolent leaders (Graen et al., 1986) and may free ride to have low work engagement. As such, we propose the following hypothesis:

*Hypothesis 4*: At the individual level, growth need strength moderates the indirect, positive relationship between benevolent leadership and work initiative through work engagement such that the indirect relationship between benevolent leadership and work initiative will be stronger when an individual’s growth need strength level is high than when it is low.

**Figure 1:**
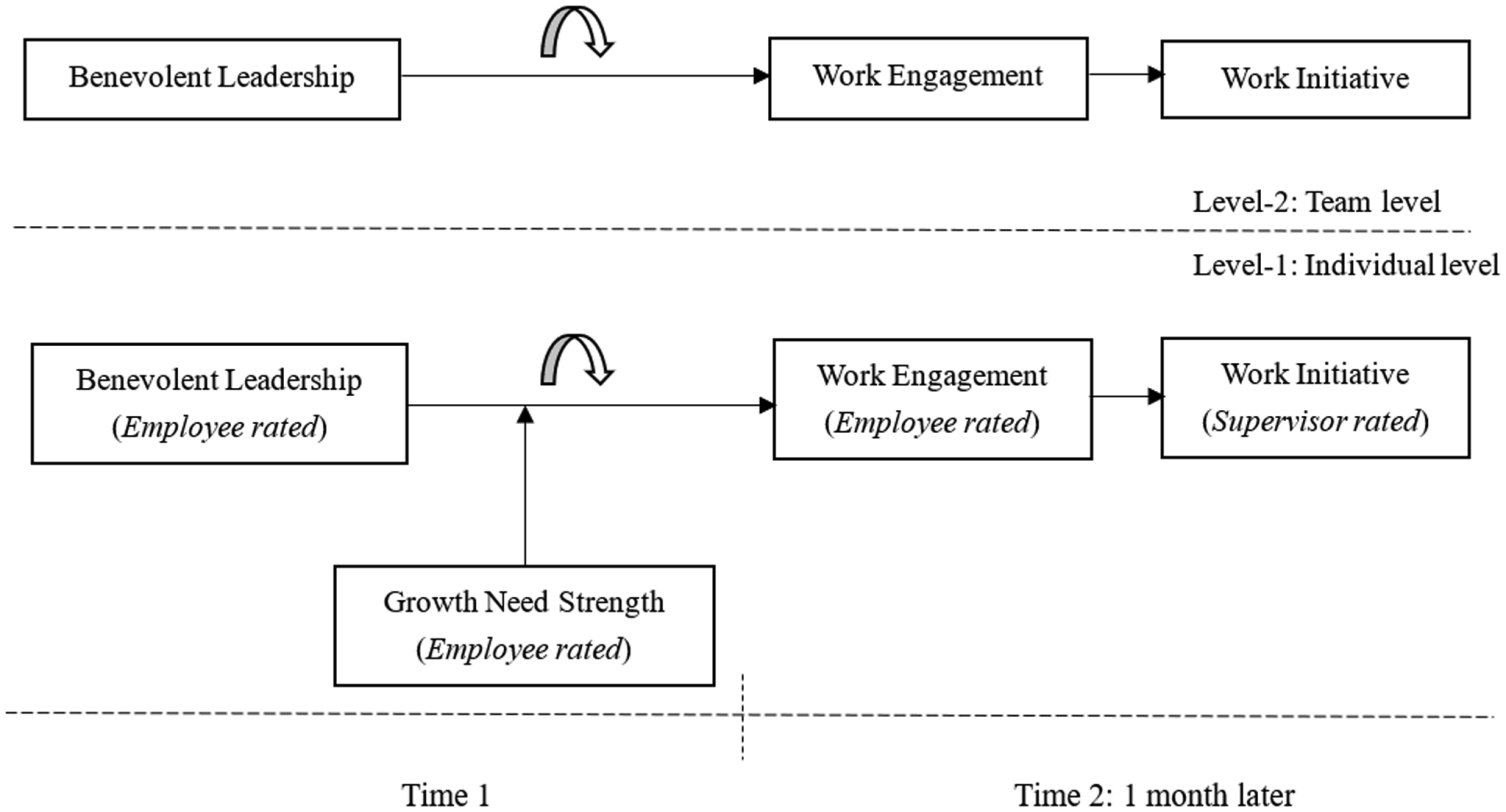
Theoretical model.

Altogether, we summarize the proposed model in Figure 1.

## Materials and Methods

### Sample and Procedure

In our study, we investigated work teams from multiple technology companies in eastern China. All participating companies produce high-technology products and services including automatic drive, smart mobile phone, communication, etc. Work teams were characterized as the departments which had three or more employees under the same leaders. We used the snowball approach to collect data from two sources (i.e., employee survey and supervisor survey) to test our multilevel structural equation model. In particular, we asked the human resources management in different companies to invite employees and their supervisors in several departments to participate in the study voluntarily. Before distributing the questionnaire, we numbered each employee and their supervisors with a unique code and sent the same coded messages to them, including the purpose of this survey and the confidentiality agreement.

Considering common method biases, we distributed a total of 680 questionnaires to team members at two stages. This provided links to the same serial number of each numbered person to match the two stages. At Time 1, employees rated benevolent leadership, growth need strength, and individual-level control variables. At Time 2, employees rated work engagement and their supervisors were asked to rate subordinates’ work initiative and team-level control variables (1 month later). The time lags are consistent with previous studies and long enough for correlations to be lower than measured for all variables at the same time (Podsakoff et al., 2012; Lin W. et al., 2018).

We sent a total of 680 questionnaires at Time 1, and 653 questionnaires were recovered. Then, we sent a total of 653 questionnaires to employees at Time 2, and 607 questionnaires were recovered. Furthermore, all supervisors provided ratings of their employees’ work initiative at Time 2. After eliminating the questionnaires that were incomplete and invalid, a total of 596 members were obtained (response rate = 87.6%), these members were from 139 teams, with the size ranging from 3 to 7 employees. For the sample at the individual level, 61.9% were under 35 years old, and 44.5% were female. The job tenure was not more than 5 years for 51.2%, and the average tenure with their supervisor was 4.37 years (SD = 2.73). Finally, 76.5% of the employees had an education level of a bachelor’s degree or below, and 56% were either married or partnered. For the sample at the team level, 34.5% of the team leaders were female, and 84.2% of the leaders had an education level of a bachelor’s degree or above.

### Measures

#### Individual-Level Variables

Because all the questionnaires used were in English, we chose the translation and back translation program to translate them into Chinese (Brislin, 1986). Unless noted otherwise, all scales used 7-point Likert scales (1 = strongly disagree; 7 = strongly agree).

##### Benevolent Leadership

We measured *benevolent leadership* using the six-item scale developed by Cheng et al. (2000) and adapted by Chen et al. (2012) within the Chinese context. A sample item is “When I make serious mistakes at work, my supervisor will give me chance to correct it.” The Cronbach’s alpha value was 0.82.

##### Work Engagement

We measured work engagement using the nine-item scale developed by Schaufeli and Salanova (2006). A sample items is “At my work, I feel bursting with energy.” The Cronbach’s alpha value was 0.89.

##### Work Initiative

Following the example of Schaubroeck et al. (2021) and Pan and Lin (2018), we adapted the *work initiative* developed by Frese et al. (1997) asking employees’ supervisors to rate their agreement with statements describing the initiative of subordinates in the past month. A sample item is “This subordinate takes initiative immediately even when others do not.” The Cronbach’s alpha value was 0.85.

##### Growth Need Strength

*Growth need strength* was measured with a six-item scale developed by Hackman and Oldham (1980). A sample item is “I enjoy stimulating and challenging work.” The Cronbach’s alpha value was 0.87.

##### Individual-Level Control Variables

We controlled for gender (0 = male, 1 = female), age (1 = less than or equal to 24; 5 = greater than or equal to 55), education level (1 = junior high school or below; 6 = doctoral degree or above), and marital status (0 = single, divorced, or widowed, 1 = married or partnered) because prior research has shown that such demographic variables are key determinants (Mauno et al., 2007; Matta et al., 2015; Ng and Lucianetti, 2016). In addition, we controlled for tenure (1 = 3 years or below; 5 = 15 years or above) because studies have also shown that tenure is correlated with work engagement (Thorsteinson, 2003; Yasin Ghadi et al., 2013). In addition, we controlled for subordinates’ tenure with their supervisor because this variable has been shown to influence supervisors’ ratings of their followers (Duarte et al., 1994) and followers’ reactions to their leaders (Haggard et al., 2011). Each subordinate answered how long he/she has worked with their current supervisor (in the number of years; Pan and Lin, 2018). Finally, because 263 of 596 employees were from the same organization, we created a dummy variable called *organizational membership* (1 = 263 respondents, 0 = remaining 333 respondents) to remove any effects that the idiosyncratic characteristic of this organization had on the data (Ng and Lucianetti, 2016).

#### Team-Level Variables

Benevolent leadership, work engagement, and work initiative could be team-level constructs. We aggregated the individual data of one team into team-level data by calculating the average values of each team, with meeting a_wg_, r_wg_, ICC (1), and ICC (2) indicators (James et al., 1993; Bliese, 2000). For benevolent leadership, ICC (1) was 0.38, ICC (2) was 0.73, the mean r_wg_ was 0.75, and the mean a_wg_ was 0.79. For work engagement, ICC (1) was 0.32, ICC (2) was 0.67, mean r_wg_ was 0.65, mean a_wg_ was 0.67. For work initiative, ICC (1) was 0.36, ICC (2) was 0.71, mean r_wg_ was 0.82, and mean a_wg_ was 0.85. Thus, all aggregation indices were found to be commonly accepted except work engagement (James et al., 1993; Brown and Hauenstein, 2005). Consistent with the argument of Giolito et al. (2020), work engagement is a psychological mechanism that is an individual construct, and a small group size will result in interrater agreement associated with the team level being less relevant (Klein and Kozlowski, 2000). Aggregation and within-group agreement indices are reported in Table 1.

**Table 1:** Aggregation and within-group agreement indices.

	ICC		r_wg_		a_wg_	
	ICC(1)	ICC(2)	Mean	Median	Mean	Median
Benevolent leadership	0.38	0.73	0.75	0.80	0.79	0.79
Work engagement	0.32	0.67	0.65	0.71	0.67	0.70
Work initiative	0.36	0.71	0.82	0.85	0.85	0.86

We also controlled for both supervisors’ gender (0 = male, 1 = female), because supervisors’ gender might influence their response to the behavior their followers conducted (Bolino and Turnley, 2003). Consistent with Giolito et al. (2020), we also controlled for supervisors’ education level (1 = junior high school or below; 6 = doctoral degree or above). Finally, we found a statistically significant relationship between the variables of our model and leaders’ gender and education level.

### Analyses

Given that Preacher et al. (2010) suggested that researchers should consider the same model at both the group and individual levels simultaneously, we conducted indirect effect tests of hypotheses using a multilevel structural equation model (MSEM). Additionally, we chose an unconflated multilevel model (UMM) instead of MSEM to test the mediation effect (Giolito et al., 2020). For the UMM approach, we grouped mean center benevolent leadership and work engagement at the individual level. At the team level, we grouped mean benevolent leadership, work engagement, and work initiative. Moreover, we used a multilevel linear model to test the moderated mediation effect because we only focused on the individual level. All analyses were run with R 4.1.2 and its packages (Bliese, 2013).

We used the following regression equation to test the moderated effect based on Pierce and Aguinis (2013) approach:

$y=\beta_{0}+\beta_{1}x+\beta_{2}w+\beta_{3}xw+\beta_{4}x^{2}+e$

As Pierce and Aguinis (2013) noted, benevolent leadership (*x*) served as a predictor and also as a moderator of the relationship between itself and the work engagement (*y*). The point of inflection occurred when $x=-\frac{\beta_{1}}{2\beta_{4}}$ (Weisberg, 2005; Pierce and Aguinis, 2013). What’s more, the growth need strength (*w*) affected not only the location of the inflection point in the benevolent leadership (*x*)—work engagement (*y*) relationship, but also the slope around the inflection point along the benevolent leadership (*x*) continuum (Pierce and Aguinis, 2013).

## Results

### Confirmatory Factor Analysis

We performed a confirmatory factor analysis to test the construct validity of the four focal variables in this study. As shown in Table 2, the hypothesized four-factor model (*x*^2^/*df* = 1.29, CFI = 0.985, TLI = 0.984, RMSEA = 0.022, SRMR = 0.029) fit the data better than other alternative models, thus providing support for the distinctiveness of the four constructs in the current study.

**Table 2:** Comparison of measurement models.

Model	*χ* ^2^	*df*	*χ*^2^/*df*	CFI	TLI	RMSEA	SRMR
Hypothesized four-factor model	443.219	344	1.29	0.985	0.984	0.022	0.029
Three-factor model	1133.596	347	3.27	0.882	0.872	0.062	0.065
Two-factor model	1724.718	349	4.94	0.794	0.777	0.081	0.079
One-factor model	2654.461	350	7.58	0.655	0.628	0.105	0.101

*N* = 596. IFI, incremental fit index; CFI, comparative fit index; RMSEA, root-mean-square error of approximation.

### Descriptive Statistics and Correlations

Tables 3 and 4 show the means, standard deviations, and correlations of the variables based on individual- and team-level data, respectively. As De Clercq et al. (2014) suggested, tenure was highly related to age (De Clercq et al., 2014). The relationship between the major variables was as expected, thereby providing initial support for all the hypotheses.

**Table 3:** Descriptive statistics and correlations, individual level.

Variables	*M*	*SD*	1	2	3	4	5	6	7	8	9	10
Gender	0.44	0.50										
Age	2.22	1.00	0.06									
Edu	3.97	0.81	0.05	−0.15								
Tenure	2.55	1.39	0.04	0.91	0.17							
MS	1.56	0.50	0.04	0.31	0.08	0.26						
TS	4.37	2.73	0.08	0.69	−0.11	0.82	0.21					
OM	0.44	0.50	−0.02	0.07	−0.38	0.06	0.04	0.03				
BL	4.29	0.90	0.02	−0.02	0.11	0.03	0.01	−0.04	−0.09			
WE	4.70	1.04	−0.04	−0.14	0.21	−0.17	0.01	−0.16	−0.09	0.42		
WI	3.75	0.75	−0.04	−0.22	0.24	−0.24	0.01	−0.24	−0.19	0.29	0.58	
GNS	4.42	1.09	0.02	−0.05	0.12	−0.08	0.06	−0.09	−0.05	0.21	0.51	0.37

*N* = 596. MS marital status; TS, tenure with leader; OM, organizational membership; BL, benevolent leadership; WE, work engagement; WI, work initiative; GNS, growth need strength. Correlations > =|0.08| are significant at = 0.1. Correlations > =|0.09| are significant at = 0.05. Correlations > =|0.11| are significant at = 0.01.

**Table 4:** Descriptive statistics and correlations, group level.

Variables	*M*	*SD*	1	2	3	4
Supervisor Gender	0.35	0.48				
Supervisor Edu	4.18	0.68	0.16			
BL	4.28	0.69	0.10	0.17		
WE	4.67	0.74	0.23	0.28	0.54	
WI	3.74	0.55	0.22	0.35	0.39	0.77

*N* = 139. BL, benevolent leadership; WE, work engagement; WI, work initiative. Correlations > |0.16| are significant at = 0.05. Correlations > |0.22| are significant at = 0.01.

### Hypotheses Development

**Figure 2:**
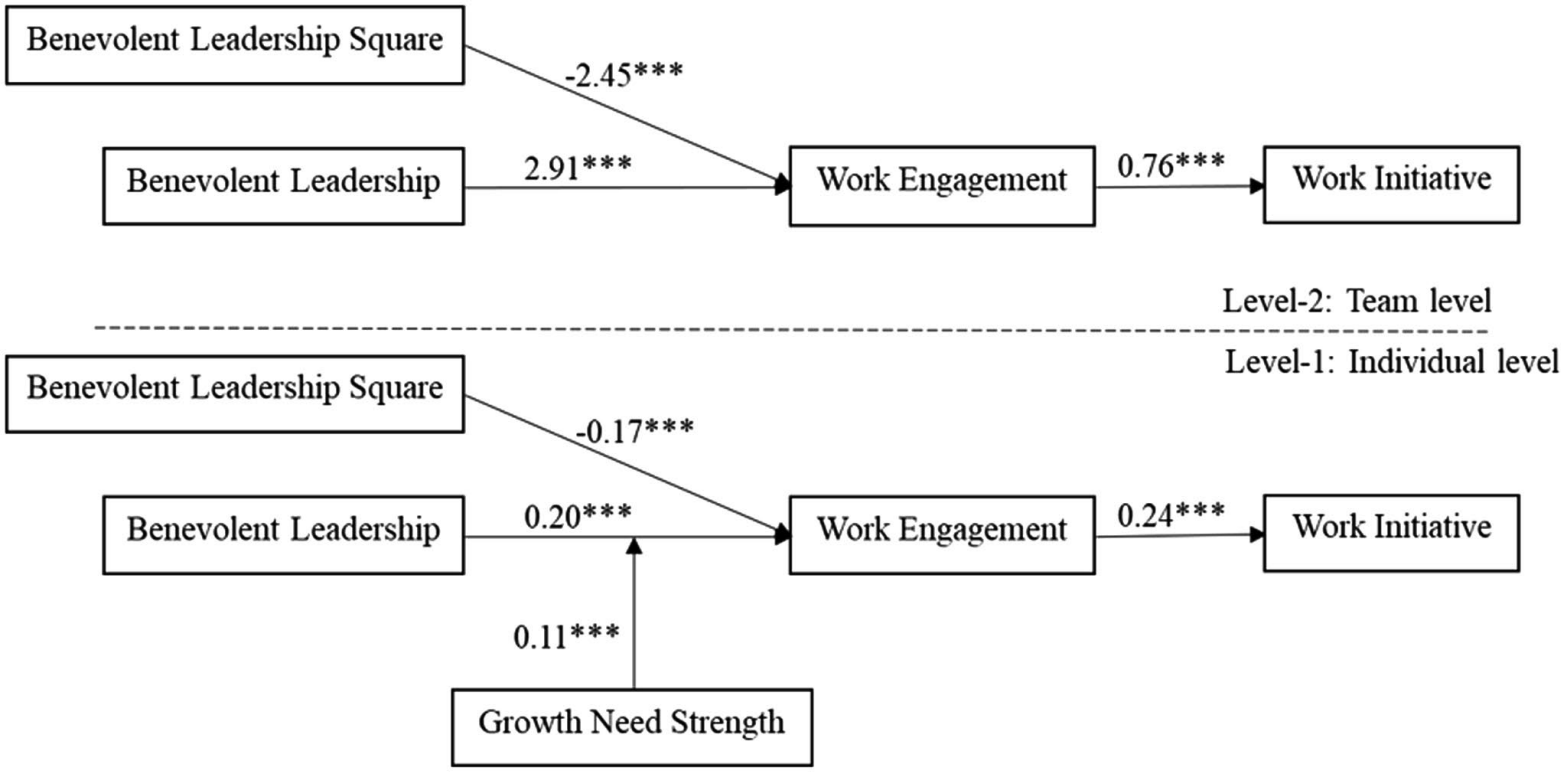
Model results.

**Figure 3:**
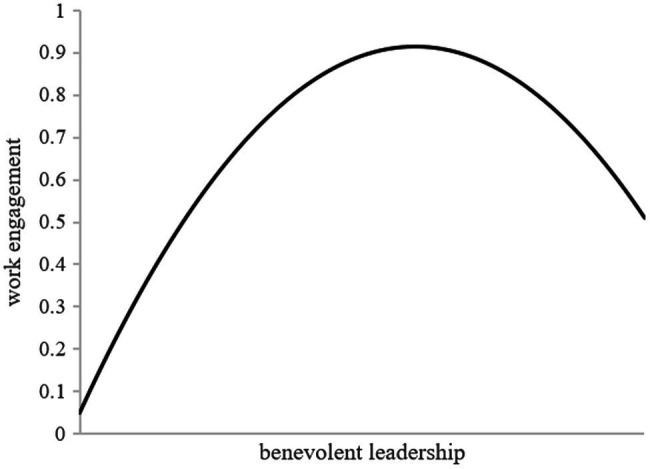
Inverted-U relationship between benevolent leadership and work engagement at the team level.

Overall model results are shown in Figure 2. Hypotheses 1a and 1b proposed that there was an inverted U-shaped relationship between benevolent leadership and work engagement at both the individual and team levels. Model 2 in Table 5 shows that when controlling for other factors, at the individual level, benevolent leadership is positively correlated with work engagement (*β* = 0.20, *p* < 0.001), and the quadratic term of benevolent leadership is negatively correlated with work engagement (*β* = −0.17, *p* < 0.001). Model 2 in Table 6 shows that at the team level, benevolent leadership is positively related to work engagement (*β* = 2.91, *p* < 0.001), and the quadratic term of team-level benevolent leadership is negatively associated with work engagement (*β* = −2.45, *p* < 0.001). Given the negative sign of the quadratic term coefficient, the result was consistent with the negative curvilinear relationship between benevolent leadership and work engagement at both the individual and team levels (Montani et al., 2020). Therefore, Hypotheses 1a and 1b were well supported, and we graphed the results at the team level, as shown in Figure 3.

**Table 5:** Unconflated multilevel model, individual level.

	Work engagement		Work initiative	
	Model 1	Model 2	Model 3	Model 4
Control variables				
Gender	−0.08	−0.06	−0.03	−0.02
Age	0.02	0.01	−0.09	−0.12
Edu	0.22^***^	0.17^***^	0.16^***^	0.13^**^
Tenure	−0.09	−0.09	−0.00	0.03
Marital status	0.07	0.03	0.06	0.07^†^
Tenure with leaders	−0.09	−0.08	−0.16^*^	−0.17^*^
Organizational membership	−0.05	−0.03	−0.23^**^	−0.26^**^
Independent variables				
Benevolent leadership		0.20^***^		0.07^†^
Benevolent leadership squared		−0.17^***^		−0.02
Mediator				
Work engagement				0.24^***^
Residual variance	0.93	0.85	0.88	0.80
*R* ^2^	0.07	0.15	0.12	0.20
*∆R* ^2^		0.08		0.08
Variance	0.93^***^	0.85^***^	0.88^***^	0.80^***^

*N* = 596 and 139 teams. Standardized coefficients are presented.

† *p* < 0.1.

* *p* < 0.05;

** *p* < 0.01;

*** *p* < 0.001.

**Table 6:** Unconflated multilevel model, group level.

	Work engagement		Work initiative	
	Model 1	Model 2	Model 3	Model 4
Control variables				
Supervisors’ gender	0.20^*^	0.12^†^	0.17^*^	0.03
Supervisors’ edu	0.26^**^	0.19^**^	0.33^***^	0.14^*^
Independent variables				
Benevolent leadership		2.91^***^		−0.40
Benevolent leadership squared		−2.45^***^		0.36
Mediator				
Work engagement				0.76^***^
Intercept variance	0.94	0.59	0.90	0.42
*R* ^2^	0.12	0.45	0.15	0.61
∆*R*^2^		0.33		0.46
Variance	0.94^***^	0.59^***^	0.90^***^	0.42^***^

*N* = 596 and 139 teams. Standardized coefficients are presented.

† *p* < 0.1.

* *p* < 0.05;

** *p* < 0.01;

*** *p* < 0.001.

Hypotheses 2a and 2b proposed that benevolent leadership had an indirect, inverted-U relationship with work initiative through work engagement at both the individual and team levels. Model 4 in Table 5 shows that work engagement is positively associated with work initiative at the individual level (*β* = 0.24, *p* < 0.001). Moreover, Model 4 in Table 6 shows that work engagement is positively related to work initiative at the team level (*β* = 0.76, *p* < 0.001). We tested the mediation effect by using a Monte Carlo simulation program at both the individual and team levels (MacKinnon et al., 2004). The indirect effect with a 95% confidence interval did not contain zero after 30,000 Monte Carlo simulations (CI_individual-level_ = [−0.06, −0.02]; CI_team-level_ = [−2.70, −1.08]), thereby supporting hypotheses 2a and 2b.

**Figure 4:**
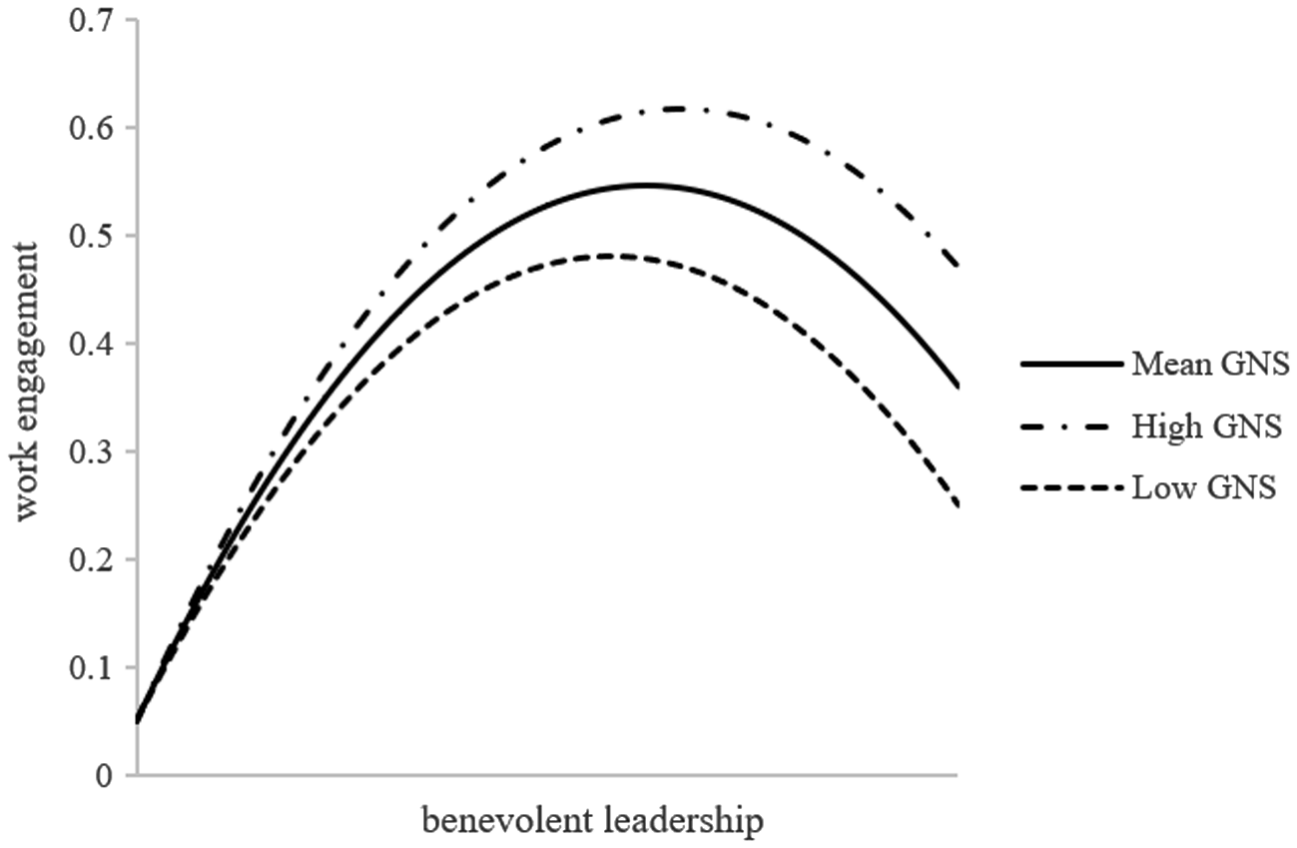
The moderating effect of growth need strength on the relationship between benevolent leadership and employees’ work engagement at the individual level.

Hypothesis 3 proposed that at the individual level, employees’ growth need strength moderates the positive relationship between benevolent leadership and work engagement. We use a multilevel linear model to verify Hypothesis 3. To reduce multicollinearity, we grand-mean centered benevolent leadership and growth need strength before calculating the interaction terms (Aiken et al., 1991). As shown in Model 1 in Table 7, the results revealed that the benevolent leadership × growth need strength interaction is significant (*β* = 0.11, *p* < 0.001). We studied the simple slope of growth need strength at high (+1 SD) and low (−1 SD) levels. Model 2 in Table 7 shows that for employees with high growth need strength, the slope term is significant and positive (*β* = 1.71; *p* < 0.001; 95% CI [1.31, 2.11]). Model 3 in Table 7 shows that for employees with low growth need strength, the slope term is significant and positive (*β* = 1.49; *p* < 0.001; 95% CI [1.12, 1.86]). We plotted a predictive model of the relationship between benevolent leadership and work engagement under high, medium, and low levels of growth need strength, as shown in Figure 4. As expected, although there is a non-monotonic relationship across all levels of growth need strength, the point of inflection where benevolent leadership starts to have a negative impact on work engagement is found at higher levels of benevolent leadership for those with a high growth need strength than for those with a low growth need strength. Thus, hypothesis 3 was supported.

**Table 7:** Moderating effect of growth need strength on the relationship between benevolent leadership and work engagement, individual level.

	Medium	High	Low
	0	+1 SD	−1 SD
*Fixed effect*			
Control variables			
Gender	−0.05	−0.05	−0.05
Age	−0.04	−0.04	−0.04
Edu	0.11^**^	0.11^**^	0.11^**^
Tenure	0.00	0.00	0.00
Marital status	−0.01	−0.01	−0.01
Tenure with leaders	−0.05	−0.05	−0.05
Organizational membership	−0.02	−0.02	−0.02
Independent variables			
Benevolent leadership	1.60^***^	1.71^***^	1.49^***^
Benevolent leadership squared	−1.29^***^	−1.29^***^	−1.29^***^
Growth need strength	0.43^***^	0.43^***^	0.43^***^
Interaction term			
Benevolent leadership × growth need strength	0.11^***^	0.11^***^	0.11^***^
*Random effect*			
Intercept	0.07	0.07	0.07
Residual	0.49	0.49	0.49
*R* ^2^	0.42	0.42	0.42

*N* = 596 and 139 teams. Standardized coefficients are presented.

** *p* < 0.01;

*** *p* < 0.001.

Hypothesis 4 proposed that the indirect inverted-U relationship between benevolent leadership and work initiative *via* work engagement would be moderated by growth need strength at the individual level. We again used the Monte Carlo simulation program (30,000 replications) to test this moderated mediation model. As shown in Table 8, the results showed that the indirect positive relationship between benevolent leadership and work initiative is stronger when growth need strength is high than when it is low (∆ = 0.10, 95% CI [0.05, 0.16]). Thus, hypothesis 4 was supported.

**Table 8:** Indirect effect through work engagement at higher and lower levels of growth need strength.

Outcome variables	Growth need strength	Indirect effects and 95% CI	Difference of effects and 95% CI
Work initiative	High (+1 SD)	0.80 (0.59, 1.03)	0.10 (0.05, 0.16)
	Low (−1 SD)	0.70 (0.49, 0.92)	

*N* = 596 and 139 teams. Indirect effects represent the mediating effect of work engagement through benevolent leadership linked to work initiative at varying levels of growth need strength CI confident intervals.

## Discussion

In our study, based on the social exchange theory (Blau, 1964; Cropanzano and Mitchell, 2005), we use a multilevel structural equation model to examine the effect of benevolent leadership on work engagement. The results of the relationship between benevolent leadership and work engagement are inconsistent with prior research conclusions (e.g., Tuan, 2018; Xu et al., 2018), that is, benevolent leadership will have an inverted-U influence on work engagement. In line with prior empirical results, work engagement has a positive impact on work initiative (Hakanen et al., 2008; Lisbona et al., 2018). However, the linkages between work engagement and work initiative at the team level are significantly stronger than those between work engagement and work initiative at the individual level (∆ = 0.52, 95% CI [0.36, 0.68]), which has also been indicated by previous studies (Tims et al., 2013; Mäkikangas et al., 2016). Although the differences existed in the association between team/individual-level work engagement and work initiative, results demonstrate an indirect, inverted-U relationship between benevolent leadership and work initiative *via* work engagement at both the individual and team levels. We also find that growth need strength serves as a moderator in the relationship between individual-level benevolent leadership and individual-level work engagement.

### Theoretical Implications

Our study offers several theoretical contributions. First, by examining the mediating role of work-related motivation state between benevolent leadership and work initiative behavior, this study contributes to the understanding of the inverted-U effect of benevolent leadership. Based on the social exchange theory, prior literature mainly highlighted the positive relationship between benevolent leadership and work engagement as well as work initiative (Chan, 2017). However, as Li et al. (2018) conceptually argued before, excessively benevolent leaders can demotivate their subordinates. According to the principle of reciprocity, there must be a punishment mechanism to prevent employees from shirking (Cropanzano and Mitchell, 2005). When employees feel that their excessively benevolent leaders will not punish them even after the serious mistake, they will free ride and reduce work engagement (Guala, 2012; Price and Vugt, 2014). Consequently, our study attempts to link benevolent leadership to work initiative by considering work engagement as the mechanism. Our results extend the understandings of the benevolent leadership–work initiative relationship by revealing that a moderate level of benevolent leadership is the best way to make employees proactive.

Second, previous studies, at most, focused on the relationship between benevolent leadership and employees’ work engagement at the individual level (Tuan, 2018; Xu et al., 2018). Theorists elucidated that employees within the same team share their work-related motivational state and proactive behavior, and this will result in team-level constructs (Brav et al., 2009; Costa et al., 2014). This study extends the prior literature and provides empirical evidence that benevolent leadership has a negative curvilinear influence on team work engagement. Arguably by definition, benevolent leaders will treat team members consistently and further encourage employees to form the team-level work-related attitude and behavior (Cheng and Wang, 2014). Data from 596 individuals and 139 teams were collected to support the probe of benevolent leadership impacting on work engagement at the team level, which verified our hypothesis. What is more, by indicating team work engagement tended to increase team work initiative at the team level (*β* = 0.76, *p* < 0.001), we find the inverted-U indirect effect of benevolent leaders on work initiative at the team level. Overall, the result enabled us to realize that when a leader showed excessively benevolent, the work initiative of the entire team would be diminished.

Third, the introduction of growth need strength as a moderator also contributes to the benevolent leadership and growth need strength literature. Our research found that growth need strength, an individual factor, acts as a crucial role in determining the effects of benevolent leadership (Shalley et al., 2009). Different employees may differ in the degree to which they value opportunities for learning, development, and growth in the workplace (Hackman and Oldham, 1980). Thus, followers’ work-related motivation will vary when they face the benevolent leader in the team. It means some followers may respond to challenge proactively, have more positive emotions than others (Graen et al., 1986; Saavedra and Kwun, 2000). The growth need strength captures this significant characteristic in our research. Although growth need strength should be taken into consideration as a critical individual characteristic in moderating the relationship between benevolent leadership and psychological state (Tiegs et al., 1992), few studies have tested the growth need strength and interaction with benevolent leadership in predicting work engagement. To fill this gap, our studies proved that growth need strength moderated the relation between benevolent leadership and work engagement. When followers have a high level of growth need strength, the point of inflection of the inverted-U relationship between benevolent leadership and work engagement is postponed (Pierce and Aguinis, 2013).

### Practical Implications

Employees who take more initiative can boost organizations’ effectiveness and job performance in the long term because those employees persist in achieving their own goals regardless of barriers in the workplace (Thomas et al., 2010; Wihler et al., 2017; Pan and Lin, 2018). Our research found that there is an inverted-U shaped association between benevolent leadership and work initiative *via* work engagement. These findings have valuable managerial implications for effectively implementing leaders’ benevolence and improving employees’ work initiative within the organization. First, our study confirmed that benevolent leadership would enhance work engagement as well as work initiative. Thus, organizations should help leaders to develop benevolence, which is crucial for cultivating employees’ sense of debt (Farh and Cheng, 2000) so as to stimulate work engagement (Breevaart et al., 2015).

Second, given that our research revealed the inverted-U effect of benevolent leadership on work initiative through work engagement, we recommend that managers should focus on the TMGT effect of benevolent leadership. Specifically, supervisors who demonstrate excessive benevolence will demotivate their employees because they omit punishment (Farh and Cheng, 2000) and drive their subordinates to lack of reciprocity norm (Cropanzano and Mitchell, 2005). Subordinates may loaf on the job and free ride to reap great profits with no punishment (Price and Vugt, 2014). This case would result in decreased employees’ work engagement. Therefore, managers should be aware that moderate levels of benevolent leadership are preferred and higher work engagement and work initiative are anticipated in this occasion.

Third, we found that growth need strength moderates the positive relationship between benevolent leadership and work engagement, which means that employees with a high level of growth need strength can delay the inflection point at which benevolent leadership has a negative impact on work engagement. Therefore, we suggest that managers should recruit, retain, and train such employees for organizations. Specifically, before hiring, managers could develop a series of questionnaires to assess growth need strength (Lin X. et al., 2018). After hiring, managers should communicate with subordinates to identify their work attitudes and growth expectations frequently (Peterson, 2005).

### Limitations and Future Research Directions

There are some limitations in the current study that must be addressed. First, although we collected time-lagged data, the use of cross-sectional data does not necessarily lead to causality. In future studies, scholars can use a more rigorous research design to verify our results. For example, researchers can use interview recording methods to further verify the relationship between benevolent leadership and work engagement. Second, we aggregated individuals’ work engagement and work initiative to form team work engagement and team work initiative, another method to assess team-level data is that entire team members collectively respond about a team-level construct, i.e., the consensus method (Kirkman et al., 2001). Findings based on aggregating individual-level data may underestimate the effects of team work engagement compared with consensus decision making (Hyatt and Ruddy, 1997; Kirkman et al., 2001). Fortunately, using consensus methods to estimate the impact of team work engagement does not change the conclusions in our research. Even so, future studies could use consensus-based measures to examine the work engagement–work initiative relation at the team level. Third, our research only discussed the moderating effect of growth need strength as an individual factor on the mechanism of benevolent leadership. In fact, the work context factor may also have an impact on subordinates’ responses to benevolent leadership (Lin W. et al., 2018). Future research could investigate whether and how work context factors (e.g., power distance orientation) impact on benevolent leadership–work engagement relation.

## Conclusion

Our study used samples from Chinese technology companies to explore the impact of benevolent leadership on subordinates’ work initiative. Unlike prior studies, which found that benevolent leadership was positively related to personal initiative (Xu et al., 2018), we found an inverted U-shaped relationship between benevolent leadership and work initiative through work engagement. Additionally, we found that the relationship between benevolent leadership and work engagement was stronger with higher (vs. lower) growth need strength such that the inflection point of the inverted-U relation is postponed.

## Data Availability Statement

The raw data supporting the conclusions of this article will be made available by the authors, without undue reservation.

## Ethics Statement

Ethical review and approval was not required for the study on human participants in accordance with the local legislation and institutional requirements. The patients/participants provided their written informed consent to participate in this study. Written informed consent was obtained from the individual(s) for the publication of any potentially identifiable images or data included in this article.

## Author Contributions

HL conceived the idea of the study and provided a theory guide. HL and SS wrote the manuscript, analyzed the data, and interpreted the results. HL, SS, and YY checked and revised the manuscript. HL and PW collected the data. HL, SS, PW, and YY discussed the results. All authors contributed to the article and approved the submitted in this article.

## Conflict of Interest

PW was employed by company Linyang Group.

The remaining authors declare that the research was conducted in the absence of any commercial or financial relationships that could be construed as a potential conflict of interest.

## Publisher’s Note

All claims expressed in this article are solely those of the authors and do not necessarily represent those of their affiliated organizations, or those of the publisher, the editors and the reviewers. Any product that may be evaluated in this article, or claim that may be made by its manufacturer, is not guaranteed or endorsed by the publisher.

## Footnotes

^1^What is the opposite of demotivation? (wordhippo.com).
